# A Modified Classification Scheme for Chagas Cardiomyopathy Incorporating Cardiac Magnetic Resonance Imaging

**DOI:** 10.7759/cureus.59900

**Published:** 2024-05-08

**Authors:** Alexandra Smith, Emilio Fentanes, Rosco Gore, Kelvin N Bush

**Affiliations:** 1 Division of Cardiology, Brooke Army Medical Center, Fort Sam Houston, USA

**Keywords:** echocardiogram (echo), treatment of cardiomyopathy, cardiac magnetic resonance (cmr), chagas cardiomyopathy, chagas

## Abstract

The public health concerns from Chagas disease warrant improved cardiovascular imaging efforts, and in this report, we review a military service member presenting with electrocardiographic and cardiac magnetic resonance imaging (CMR) findings that recognized a Chagas dilated cardiomyopathy. We present an updated Chagas staging classification incorporating CMR to increase diagnosing cardiomyopathies.

## Introduction

Chagas disease and the rising public health concerns warrant improved cardiovascular imaging availability and treatment management to address the endemic increasing morbidity [1]. Current Chagas disease classification schemes published from 2018 do not incorporate cardiac magnetic resonance imaging (CMR) into the staging classification scheme [1]. We present a case that highlights the role of CMR in the presentation of a military service member diagnosed with a Chagas cardiomyopathy and propose a modified Chagas imaging classification scheme to aid in classifying dilated cardiomyopathies, fibrosis, or inflammatory changes in this population.

## Case presentation

**Figure 1:**
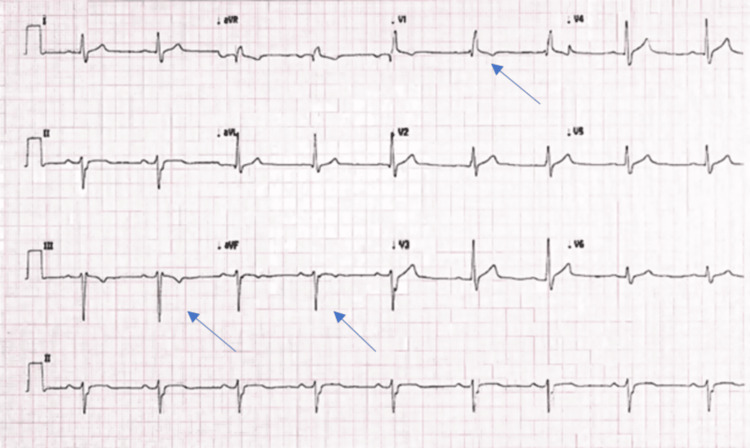
A 12-lead electrocardiogram of the patient demonstrates normal sinus rhythm, first-degree AV delay (PR interval 225 msec), right bundle branch block, and a left anterior fascicular block. AV: atrioventricular

An 18-year-old asymptomatic military basic trainee was screened positive for Trypanosoma cruzi with an enzyme-linked immunosorbent assay and confirmed by the Center for Disease Control with both a reactive enzyme immunoassay and a trypomastigote excreted-secreted antigen-positive immunoblot. A native of South Texas, he reported having frequent and common exposure to many triatomine bugs without any clear history of chagoma or bites. The patient was referred for cardiovascular evaluation, and an abnormal electrocardiogram revealed sinus bradycardia, first-degree atrioventricular delay, a right bundle branch block, and a left anterior fascicular block (Figure 1). The review of systems was negative for any cardiovascular symptoms of dyspnea, chest discomfort, heart failure, or gastrointestinal symptoms.

**Figure 2:**
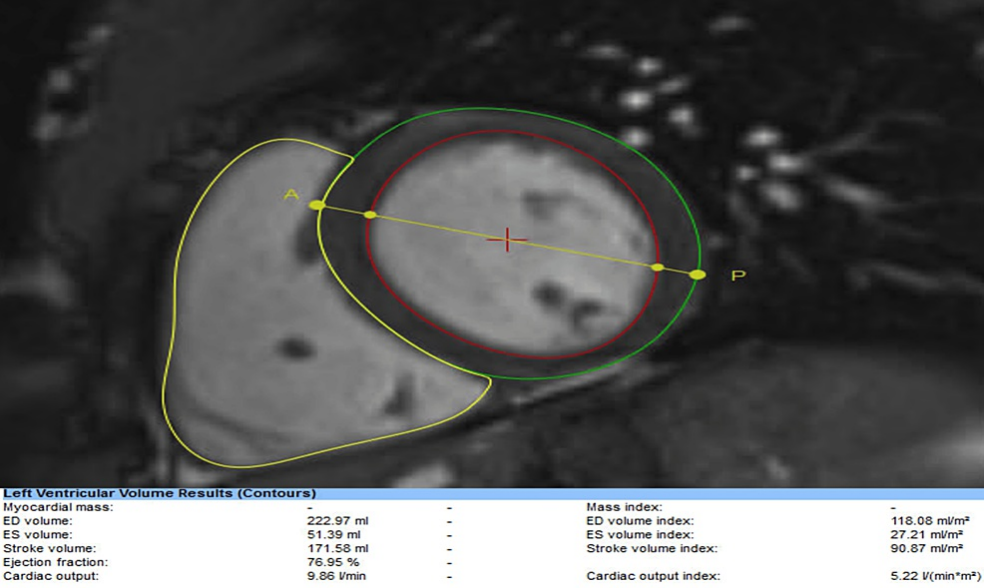
CMR demonstrating dilated left ventricle in case presentation CMR: cardiac magnetic resonance

A 24-hour ambulatory monitor revealed no arrhythmias. A transthoracic echocardiogram demonstrated a preserved left ventricular ejection fraction, normal diastolic function, no regional wall motion abnormalities, or significant valvular disease. CMR was performed given the electrocardiogram findings to evaluate specifically for myocardial infiltration, inflammation, or chamber size derangements for prognostication and military disposition purposes in the setting of the abnormal electrocardiogram findings. CMR demonstrated a left ventricular ejection fraction of 76%, mild left ventricular dilation (118.08 ml/m2, Figure 2), and normal right ventricular systolic function and size. There was no evidence of infiltration or inflammation of the myocardium by gadolinium enhancement with normal T1 relaxation times and no evidence of focal edema by T2-weighted images.

Management

The service member’s exposure history, positive serologies, electrocardiogram abnormalities, and CMR identifying left ventricular dilatation were consistent with Chagas dilated cardiomyopathy. Department of Defense enlistment standards prohibit conditions of cardiomyopathies, dilation, or cardiomegaly from entering military service, and the member was subsequently discharged and offered medical therapy (Table 1). The patient was referred to a local provider for long-term care and has not followed up with our medical facility.

**Table 1:** Expert consensus recommendations for antitrypanosomal treatment in Chagas disease, adapted from the 2018 ACC/AHA Scientific Statement Update of the Current Clinical Knowledge and Management of Chagas ACC: American College of Cardiology; AHA: American Heart Association; CM: cardiomyopathy

Treatment should be given
Acute infections, regardless of the mechanism
Women of childbearing age after a negative pregnancy test
Accidental high-risk contaminations
All cases of reactivation of T.cruzi infection
Pediatric patients with chronic phase (children <18 years of age)
Treatment could be offered
Indeterminate form in patients >18 years of age
Chronic cardiomyopathy form; not in advanced stages after shared decision-making
Treatment should NOT be given
Patients with established dilated cardiomyopathy

## Discussion

Three phases recognize Chagas disease: the acute phase, the chronic indeterminate phase, and the determinate phase (evidence of cardiac or gastrointestinal involvement). The acute phase is symptomatic for 5% of infected individuals, with low mortality rates of less than one per 2,500 infections. Fifty percent of infected patients go on to be categorized in the indeterminate phase chronically without progressing or developing [1]. Of those previously infected in the indeterminate phase, 30-40% do progress to manifest end-stage heart disease [2].

Per the current Brazilian Consensus Classification and American College of Cardiology/American Heart Association classification schemes, our patient was classified as Stage B1 and Stage B, respectively. In addition, his risk score of 2, according to Stanaway et al., corresponds to only a 10-year risk of 10% (Table 2) [3].

**Table 2:** Risk of death based on six prognostic factors adapted from Stanaway et al. Our patient was low-risk, with a score of 2 based on echocardiogram data. NYHA: New York Heart Association

Risk factors with the scoring system	Risks scores according to the points system	10-year mortality risk %
NYHA class III or IV (5 points), cardiomegaly on chest x-ray (5 points), segmental or global wall motion on echocardiography (3 points), nonsustained ventricular tachycardia (3 points), low QRS voltage (2 points), male sex (2 points)	Low Risk (0-6 points)	10%
	Intermediate Risk (7-11 points)	44%
	High Risk (12-20 points)	84%

We propose that CMR be incorporated into the Chagas classification scheme used in the Latin American Guidelines and other staging systems for Chagas cardiomyopathy (Table 3) [4] in order to aid in better identifying subclinical structural heart disease not apparent by echocardiography, recognizing fibrotic or inflammatory myocardial involvement, which is prognosis-altering (Table 4), and diagnosing early involvement of cardiomyopathy, which could benefit from earlier anti-parasitic therapy.

**Table 3:** Recommended incorporation of CMR into the Chagas cardiomyopathy staging classification Adapted from the 2018 ACC/AHA classification scheme. Our modifications are in gray. ACC: American College of Cardiology; AHA: American Heart Association; HF: heart failure; LGE: late gadolinium enhancement; LV: left ventricle, LVEF: left ventricular ejection fraction; CMR: cardiac magnetic resonance imaging

	Symptoms	Electrocardiogram	Echocardiogram	CMR
Stage A	No HF signs/symptoms	Normal ECG findings	Normal	Normal LVEF >50%, no LV cavity dilation, normal T1/T2 findings, no LGE
Stage B1	No HF signs/symptoms	+/- Arrhythmias or conduction disorders	Abnormal echocardiogram, LVEF > 45%	Normal LVEF >50%, LV cavity dilated, normal T1/T2 findings, no LGE
Stage B2	No HF signs/symptoms	+/- Arrhythmias or conduction disorders	Abnormal echocardiogram, LVEF < 45%	LVEF <50%, LV cavity dilated, normal T1/T2 findings, no LGE
Stage C	Compensated HF signs/symptoms	+/- Arrhythmias or conduction disorders	Abnormal echocardiogram, LVEF < 45%	LVEF <50%, LV cavity dilated, +T1/T2 abnormality, no LGE abnormality
Stage D	Refractory HF	+/- Arrhythmias or conduction disorders	Abnormal echocardiogram, LVEF < 45%	LVEF <50%, LV cavity dilated, +T1/T2 abnormality, + LGE abnormality

**Table 4:** Patients with indeterminate disease and normal electrocardiogram and echocardiogram studies may have subclinical disease of the myocardium that is evident on CMR The table is adapted from Torreao et al. reviews the presence of T2-weighted hyperintensity (edema), T1 early gadolinium enhancement (hyperemia), or late gadolinium enhancement (fibrosis) according to different clinical phases. CMR: cardiac magnetic resonance imaging; IND: indeterminate phase; CPND: cardiac phase without LV systolic dysfunction; CPD: cardiac phase with LV systolic dysfunction (LVEF <=55%)

	IND	CPND	CPD
T2-weighted hyperintensity (edema)	3/16 (18.8%)	16/17 (94.1%)	21/21 (100%)
T1 early gadolinium enhancement (hyperemia)	3/12 (25%)	12/13 (92.3%)	16/17 (94.1%)
Late gadolinium enhancement (fibrosis)	2/16 (12.5%)	16/17 (94.1%)	21/21 (100%)

CMR provides excellent spatial resolution and can overcome limited transthoracic imaging windows. Increased resolution improves the ability to detect subtle wall motion abnormalities in the typical apical distributions found in Chagas cardiomyopathy. Further steady-state free precession and dark blood imaging have superior sensitivity and accuracy than echocardiography. CMR also allows for more reproducible chamber quantification and ejection fraction calculations [5].

Fibrotic myocardium can be inferred from the presence of myocardial delayed enhancement (MDE) using T1-weighted CMR images. The pattern of MDE found in Chagas cardiomyopathy varies from patterns typically seen in coronary disease (subendocardial and transmural) to those more common in inflammatory cardiomyopathies (mid-myocardial and sub-epicardial), but the distribution commonly involves the basal inferolateral wall and apical segments [6]. Myocardial fibrosis can be a nidus for re-entrant ventricular arrhythmias, which are a common cause of death in Chagas cardiomyopathy [7]. The presence of MDE is an independent marker for adverse events in Chagas cardiomyopathy [8]. T1-weighted CMR images can also be used to determine tissue-native T1 times. Elevated values can be seen in both inflammation and fibrosis.

We suggest using CMR when available in select patients with indeterminate disease as an additional imaging modality for occupational purposes, unclear correlatory symptoms, cardiovascular symptoms not proportionate with echocardiogram information, and poor quality echocardiogram available information to influence the role of initiating anti-parasitic therapy and halt the progression of cardiac fibrosis and remodeling. We believe this could be one mechanism that could potentially decrease and impact long-term cardiovascular morbidity and mortality, and additional prospective studies are needed to confirm this.

## Conclusions

Chagas disease disproportionately impacts those from lower socioeconomic backgrounds, and approximately 30% of infected patients will develop Chagas cardiomyopathy. Patients with cardiac involvement demonstrated by ECG changes without echocardiographic changes could benefit from CMR imaging in select cases for the benefit of increasing sensitivity for fibrotic, inflammatory, or chamber dilatation, information that could be used to diagnose and provide prognostic information related to Chagas cardiomyopathy. Additional studies are needed to evaluate the impact of medical therapy in the earlier stages of Chagas infections. 
